# Effects of automated teller machine service quality on customer satisfaction: Evidence from commercial bank of Ethiopia

**DOI:** 10.1016/j.heliyon.2023.e19132

**Published:** 2023-08-14

**Authors:** Abibual Getachew Nigatu, Atinkugn Assefa Belete, Getnet Mamo Habtie

**Affiliations:** a Department of Accounting and Finance, Samara University, Semera, Ethiopia; b Department of Economics, Samara University, Semera, Ethiopia; c Department of Statistics, Samara University, Semera, Ethiopia

**Keywords:** Afar region, ATM, Customer satisfaction, Service quality

## Abstract

The banking industry has seen the emergence of numerous service delivery channels, automated teller machines (ATMs), telephone banking, and online banking. Global financial systems and mature competition have been compelled to research the importance of consumer satisfaction. Thus, this study aimed to examine the effects of automated teller machine service quality on customer satisfaction in commercial banks of Ethiopia in the Afar regional state, Northeast Ethiopia. To this end, cross-sectional data were collected through a questionnaire from a sample of three hundred forty-six (346) ATM users selected from Semera-logia City, Asyaita, and Awash Town. To identify the dimensions of automated teller machine service quality and their relationship with customer satisfaction, confirmatory factor analysis and structural equation modeling (SEM)) through SPSS and AMOS 23.0 data analysis software were used. The findings indicate that convenience, reliability, ease of use, fulfillment, and security/privacy of ATM service quality dimensions are positively and significantly associated with customer satisfaction. The results of this study can help banks' management improve the quality of their ATM services to increase overall customer satisfaction. To ensure continued customer satisfaction, banks are encouraged to make the ATM service more convenient, reliable, user-friendly, secure, and fulfilling. They should also constantly update and differentiate their ATM service quality aspects to build a competitive advantage and boost their profitability.

## 1 Introduction

Effective and cost-effective methods are needed to survive and generate profits in today's fast-paced, fiercely competitive global market. These revenues can then be used to fuel the organization's expansion. It is evident that customers are more significant stakeholders in many firms, and marketing management places a high focus on ensuring their pleasure. In many service sectors, technology is one of the most important forces behind increased client attraction, improved service delivery, and improved transaction execution [1]. The banking industry is regarded as the heart of global business in the era of advanced technologies. To expand the competitive market share, technological innovation improves the efficiency of banking operations and systems. The use of technology is helping the banking sector expand quickly. Information technology has revolutionized the banking sector over the past few decades and given banks a way to serve their consumers with products and services of high quality [2].

A banking company can only set itself apart from rivals by offering top-notch services. As a result of technological advancements, businesses may now offer improved services that satisfy clients [3]. Conventional banking systems are being replaced by electronic-based business models, and most banks are reevaluating their business process designs and customer relationship management strategies. Due to the low cost of comparing options in online contexts, researchers have suggested that electronic service quality is a major factor in differentiating service offers and creating a competitive advantage [4].

To be competitive, banks are expanding their electronic service options, such as SMS, mobile banking, internet banking, and ATMs. The trend in the banking industry changed from a cash economy to a cheque economy, which then shifted to a plastic card economy [5]. Clients primarily used automated teller machines out of all of these, which are self-service technology devices, and are the most commonly used electronic banking product. Using their cards in public places, customers of banks can access financial transactions like cash withdrawals, prepaid mobile phone credits, fund transfers between accounts, and checking account balances without the assistance of a bank teller [6].

The Ethiopian banking sector is also adopting this ICT-based service to clients to improve operational efficiency by lowering operating costs, which would ultimately boost client satisfaction and profitability. The state-owned Commercial Bank of Ethiopia (CBE) is the first bank in Ethiopia to introduce ATM service for a long time ago as part of ensuring service excellence by reducing waiting time, errors, and costs, thereby improving client satisfaction. Even though ATM service has a great significance in improving customer satisfaction, the services in Ethiopia are challenged by inappropriate infrastructure, unavailability of competent and skilled employees in the banks, and related problems. To encourage further ATM service expansion in the country, a better understanding of ATM service quality dimensions and client satisfaction is critical [7]. Customer impressions of ATM service quality are gauged by the ATM's ability to perform these tasks to their satisfaction. Researchers and corporations have been interested in the relationship between customer satisfaction and service quality because certain studies have shown that it exists [8].

Researchers such as [[9], [10], [11], [12], [13], [14], [15], [16]] conducted on ATM service quality in different parts of the world. In Ethiopia, previously scant research has been done on ATM service quality and its effects on customer satisfaction in Ethiopia, such as Embiale [17] conducted a study on the effect of automatic teller machine service quality on customer satisfaction: the case of the Commercial Bank of Ethiopia in Hawassa city, and conclude that aspects of ATM service quality, such as reliability, convenience, user-friendliness, security, and responsiveness, positively and significantly affect customers' satisfaction. Tewodros and Debela [18] studied Factors affecting customers' satisfaction towards the use of automated teller machines (ATMs): A case in a commercial bank of Ethiopia, Chiro town, responsiveness, efficiency, appearance, reliability, and convenience of ATMs have a significant and positive influence on customers’ satisfaction. Tadesse and Bakala [19] also conducted a study on the effects of automated teller machine service on client satisfaction in the Commercial Bank of Ethiopia and found a positive relationship between tangibles, reliability, responsiveness, empathy and assurance, and client satisfaction.

However, those studies were complicated by some issues. The selection of quality dimensions in previous studies often lacked a strong theoretical foundation. As a result, the dimensions for Automated Teller Machine (ATM) quality were chosen from existing literature on electronic quality without considering the unique attributes and aspects of ATM service quality. The studies have also neglected a crucial aspect of ATM quality such as fulfillment which has been suggested as a major ATM service quality dimension [9,20]. Also, the convenience sampling methodology adopted in prior studies and the limited geographic scope of those studies could affect the generalizability of the results which requires the need for a more comprehensive study. Overall, the existing literature falls short of providing a comprehensive understanding of the effect of ATM service quality on customer satisfaction, as it fails to consider the unique attributes of ATM services, and explores the holistic nature of ATM services.

An investigation into the dimensions of ATM service quality and their relationship with customer satisfaction is crucial due to the rising popularity of ATMs in retail banking, and this study is intended to address the identified gaps and enhance understanding of the unique characteristics of ATM services, shed light on the dimensions of service quality that customers value, and provide valuable insights for banks to improve customer satisfaction in the specific context of banking through ATM. The survey will give bank management a thorough understanding of how to manage client expectations and raise ATM user satisfaction. The objectives of this study are therefore to examine the dimensions of perceived ATM service quality evaluate the relative importance of these dimensions in predicting customer satisfaction based on the perceptions of customers and provide insight into ATM service quality in a region often understudied in academic discourse and inquiry.

## 2 Literature review

### 2.1 ATM service quality and customer satisfaction

ATMs are technological tools that enable users to deposit, withdraw, and transfer money, pay bills, and carry out other financial activities without a branch employee or teller's assistance [13]. From the aforementioned, it is claimed that an ATM is the electronic equivalent of a traditional banking hall. Customers visit an ATM to conduct financial transactions, such as withdrawals, deposits, or balance checks, just as they would have done in a traditional banking hall.

Service quality is an important factor that influences how attractive a service provider is to customers [21]. If a company provides high-quality products that fulfill customer needs, it initially ensures customer satisfaction. Therefore, to enhance customer satisfaction, service providers should enhance the quality of their services. In simpler terms, service quality and customer satisfaction have a positive relationship, where the quality of service is the primary factor that determines customer satisfaction [[22], [23], [24]]. Providing high-quality customer service is crucial for establishing and maintaining a positive rapport with customers in the traditional banking industry [22]. In this sector, delivering exceptional customer service plays a vital role in fostering positive relationships with clients [25]. Specifically, concerning ATM services, service quality refers to the customer's general evaluation and judgment of the quality of services received through the ATM channel [26].

Customer satisfaction is of utmost importance for both customers and banks. It pertains to evaluating how well a bank's products and services meet or exceed customer expectations [27]. Customer satisfaction refers to the state in which customers feel adequately rewarded or compensated in a buying situation in exchange for a particular cost [28]. Farris, Bendle [29] also described that customer satisfaction refers to the extent to which customers are happy with the products and/or services provided by a business. According to Habte and Mesfin [30], customer satisfaction refers to a response that is specific to a particular focus and time. It is closely associated with meeting the needs of clients and is recognized as a significant factor in influencing their future purchasing decisions [31]. When discussing client satisfaction, it can be described as an individual's level of pleasure or discontentment derived from comparing the perceived performance of a product with their expectations [32]. Customer perceptions of ATM service quality are determined by how effectively the ATM meets its expectations and fulfills its desired tasks.

According to the literature, ATM service quality dimensions are multidimensional [15,[33], [34], [35]]. Several aspects of ATM service quality, including reliability, convenience, security and privacy, ease of use, and fulfillment, were recognized by Ref. [33]. This study tried to examine the ATM's service quality dimensions such as reliability, convenience, security and privacy, ease of use, and fulfillment. A review of these dimensions and their alleged connection to customer satisfaction is given in the next section.

***Convenience*** is the state of having work simple and hassle-free [36]. Narteh [33] described convenience as the location of the ATM and involves the availability of services to clients around the clock. ATMs are conveniently positioned at bank branches and other locations, like malls and colleges. Customers can withdraw money from other ATMs for a minimal price because the bank's ATM card is compatible with systems used by other banks. It reduces the hassle of utilizing ATMs and has been found to positively correlate with client satisfaction. It is less inconvenient to conduct financial transactions when ATMs are nearby, as this eliminates the need to travel far [12]. According to Olusanya and Fadiya [11], customer satisfaction has been found to positively correlate with convenience, which is the most frequently used factor of ATM service quality.

Hypothesis 1 Convenience of ATM service has a significant positive relationship with customer satisfaction.

***Reliability*** is the ability to perform the desired service exactly and dependably [37]. Wolfinbarger and Gilly [38] claimed that reliability is a good predictor of client satisfaction in electronic channels. According to Ennew, Waite [39], reliability can be viewed as the degree to which customers can rely on the service that the company has promised. The reliability dimension is essential because it incorporates the active competency to carry out all the contracted services consistently and precisely. Reliability in the context of an ATM setting refers to the capacity of the device to operate continuously and deliver services that are constant and error-free. According to Ogbeide [10], reliability is a crucial ATM service quality dimension that affects client satisfaction. The literature that is currently available has also demonstrated that in the banking industry, reliability and customer satisfaction are positively correlated [[40], [41], [42], [43]].

Hypothesis 2 Reliability of ATM service has a significant positive relationship with customer satisfaction.

**Ease of Use:** Since some customers may feel threatened by technology, one would anticipate that ATMs would be designed to speed up the transactional process for users [44]. Ease of use is the extent to which the prospective user anticipates the target system to be free of effort, according to Davis, Bagozzi [44]. Chong, Ooi [45] argued that people are more inclined to use an electronic banking system if they think it's easy and stress-free. The term "concept" in this study refers to the extent to which an ATM service provides a hassle-free transaction for the user. An important factor in determining the acceptance and utilization of different corporate information technologies, such as online banking, is the ease of use [46]. The ease of use was identified by Refs. [9,47,48] as an essential ATM quality dimension.

Hypothesis 3 Easy use of ATM service has a significant positive relationship with customer satisfaction.

***Fulfillment:*** The extent to which the website's guarantees about order delivery and item readiness are upheld is what matters [20]. Wolfinbarger and Gilly [38] stated that the fulfillment of websites has a significant effect on overall quality, satisfaction, and loyalty goals. In the ATM context, the study employed fulfillment quality to gauge how well the ATM delivers results that live up to client expectations One could also indicate the amount given to clients per transaction, the transactional fees charged by the ATM, and the validity of the notes handed by the ATM (to eliminate counterfeits). Fulfillment was found to be the primary determinant of customer satisfaction for ATM service quality [13].

Hypothesis 4 Fulfillment of ATM service has a significant positive relationship with customer satisfaction.

***Security and Privacy:*** Customers should receive protection and privacy from an ATM. While privacy is the defense of personal information, security includes protecting clients against fraud and financial loss [49]. Security was described by Casaló, Flavián [50] as the technical guarantee that the legal requirement and practices protecting privacy will be properly met. The adoption of Internet banking in Vietnam was found to be influenced by security and privacy [45]. Every client expects their banks to protect their personal information and money. Khan [15] concluded that security and privacy were important aspects of the ATM service quality.

Hypothesis 5 Security and privacy of ATM service have a significant negative relationship with customer satisfaction.

**Fig. 1:**
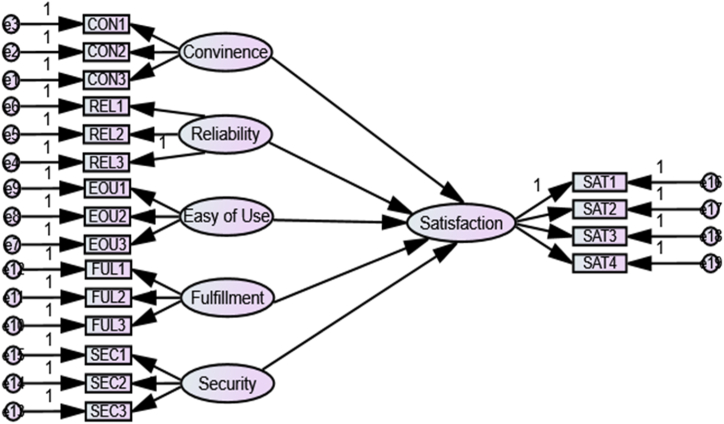
Conceptual model of ATM service quality and customer satisfaction.

Based on the previously studied literature that is already in existence, the current study predicts that the key aspects of ATM service quality that will affect customer satisfaction are reliability, convenience, security and privacy, ease of use, and fulfillment. Based on the previous discussions, the study suggests a framework that directs the ongoing research, as shown in Fig. 1.

## 3 Research methods

### 3.1 Study area

This study was conducted in Samara-Logia city, Asayita, and Awash town located in Afar regional state, eastern Ethiopia. Currently, the region is growing into a place of considerable social, economic, and political activities with the establishment of colleges, a lot of commercial banks, a university, factories, modern shops, investment projects, and other significant financial institutions.

### 3.2 Research design

A research design is an overall strategy for the study that outlines the precise steps a researcher should take to answer the study's questions and accomplish its objective [51]. The study's research design strategy is designed based on the objectives of the study. To achieve the objectives of the study, both descriptive and explanatory research methods are employed to describe and evaluate how automated teller machine service quality affects customer satisfaction. In addition, this study collected data over a specific period to look into the effect of ATM service quality on customer satisfaction in addition to its descriptive and explanatory nature. As a result, our study used the cross-sectional data type for this investigation based on the time horizon dimension of research design classification.

### 3.3 Sampling techniques and sampling size

A multi-stage sampling technique was used in this study. In the first stage, from the regional state of Afar, two towns (Awash and Asayita) and one city administration (Samara-logia) were selected purposively because there are large numbers of ATM users in those areas. In the Second stage, the sample size from each selected area was determined proportionally to the number of registered ATM users in the Commercial Bank of Ethiopia operated in each town and city. Finally, 386 ATM users were selected based on the simple random sampling method. To derive the sample size, the study used a [52] simplified formula by considering a sample error of **5%.**

### 3.4 Data source and collection procedure

The sources of data for this study are both primary and secondary data sources for the achievement of the objectives. The primary data were obtained from ATM users of commercial banks of Ethiopia (CBE) in selected towns and a city through structured questionnaires. The data were collected from October 2022 to December 2022. The secondary sources were obtained from published and unpublished materials and annual reports of the bank.

### 3.5 Measurement of instruments

The survey questionnaire for the study consists of demographic variables and five constructs in the research model. Demographic variables for acquiring information about participants consisted of gender, age, marital status, educational background, and occupation. Other than that, to identify respondents who have experience in using conventional banking services and ATMs a screening question was included in demographic questions. The survey items were assessed based on a five-point Likert-type scale, where 1 indicates strongly disagree and 5 indicates strongly agree. The construct, reliability, convenience, security and privacy, ease of use, and fulfillment were assessed by 3 items, and lastly, customer satisfaction was assessed by 4 items, adopted from Refs. [13,15,34,53] with some modification.

### 3.6 Statistical technique

The structural equation modeling (SEM) approach using Amos was employed for data analysis. This study consists of 15 latent variables, which can be deemed complex; therefore, SEM is considered appropriate for dealing with complex research models with larger numbers of latent variables [54]. The reliability, convenience, security and privacy, ease of use, and fulfillment of ATM service quality were the exogenous variables, and customer satisfaction was the endogenous variable.

### 3.7 Normality test

After gathering data, the participants' answers were organized, stored, and examined using SPSS, the statistical software. To determine whether the data followed a normal distribution, the kurtosis and skewness were assessed using two tests called the Shapiro-Wilk and Kolmogorov-Smirnov tests. The findings of the normality tests showed that they were not significant, meaning that the p-values obtained were greater than 0.05. This suggests that the distribution of the data was indeed normal. Given that our null hypothesis assumes a normal distribution, we do not reject the hypothesis when the p-value is greater than 0.05.

### 3.8 Common method variance

According to Aslam, Arif [36], common method variance (CMV) presence in the study has to be detected first before examining the measurement model to prevent any bias. To detect any bias, this study used Harman's single factor test, which stated that if the variance is less than 50%, then it indicated no CMV issue. In this study, it is indicated that the percentage of variance is 45.39%, which indicates no presence of data bias in this study.

### 3.9 Ethical consideration and consent to participate

The College of Business and Economics at Samara University provided ethical clearance. The confidentiality of the data was protected by removing respondents' identifiers, such as names, from the data collection format.

Finally, those who were willing to engage in the study and were in the sampled city and towns provided verbal informed consent. Moreover, the results were recommended to be disseminated by the responsible bodies who were involved in the Commercial Bank of Ethiopia.

## 4 Results

Out of the 386 questionnaires distributed to selected ATM users, 26 questionnaires were not returned for various reasons. In addition, 14 questionnaires were not appropriately completed by the respondents. Therefore, 346 questionnaires were analyzed, which accounted for a response rate of 89.6%.

### 4.1 Demographic profile of respondents

Table 1 presents the background profile of the users of ATM services provided by the Commercial Bank of Ethiopia in Awash and Asayita Towns and Samara-logia city administration. Out of the total completed ATM user survey in the study area, about 73.4% were male and the remaining 26.5% were female customers. Among the total samples, the majority age group of ATM users (50.3%) was 27–35 years, 19.9% were 36–50 years age group, whereas, 10.1% were 18–26 and 19.7% of them were over 51 years. Concerning marital status, the dominant 70.8% of samples are single, followed by married 19.7%, divorced 6.9%, and widowed 2.6% (Table 1).

**Table 1:** Background of Respondents (ATM users).

Variables	Frequency	Percentage	Variables	Frequency	Percentage
**Gender**			**Occupation**		
Male	254	73.4	Student	50	14.5
Female	92	26.5	Unemployed	97	28.0
Total	346	100	Salaried	137	39.6
**Age**			Businessman	62	17.9
18–26 years	35	10.1	Total	346	100
27–35 years	174	50.3	**Years being a CBE customer**		
36–50 years	69	19.9	below 1 year	34	9.8
Above 51 years	68	19.7	1–2 years	43	12.4
Total	346	100	3–4 years	29	8.4
**Marital status**			5–6 years	144	41.6
Single	245	70.8	Over 6 years	96	27.7
Married	68	19.7	Total	346	100
Divorced	24	6.9	**Years being an ATM user**		
Widowed	9	2.6	below 1 year	12	3.5
Total	346	100	1–2 years	43	12.4
**Education**		.	3–4 years	50	14.5
Illiterate	2	0.6			
			5–6 years	128	37
Secondary Education	93	26.9	Over 6years	113	32.7
Certificate and Diploma Diploma	130	37.6	Total	346	100
Degree and Above	121	35.0			
Total	346	100			

Regarding their levels of education, the majority of the total sample respondents 72.6% of them had completed a diploma and above or higher qualifications, with 26.9% having secondary education, and 0.6% of them were illiterate. Of the total sample respondents, 39.6% were salaried individuals, 28% were unemployed individuals, and 17.9% and 14.5% of the sample of ATM users were businessmen and students respectively (Table 1).

Regarding customer's experience in using the conventional banking service, from the total ATM users, 41.6% of them have 5–6 years of experience, 27.7% of them have over 6 years of experience, 12.4% of them have 1–2 years of experience, 9.8% of them have an experience of below one year, and 8.4% of them have 3–4 years of experience in the bank. Concerning using ATM service, out of the total sample respondents, about 37% of them have 5–6 years of experience, 32.7 of them have over 6 years of experience, 14.5% of them have 3–4 years of experience, 13.3% of them have 1–2 years of experience, and 3.7% of them have an experience of below one year in using ATM service from the bank according to Table 1. This indicates that, on average, most of the ATM banking users in the study area have an experience of more than three years. The longer period of the users of the ATM service served contributes to evaluating better how the ATM service of the bank is effective and efficient. Besides that, it helps the researcher to gain responses, which are most likely expected to be reliable information as a customer rating their level of satisfaction or dissatisfaction concerning services received from the bank in the study area.

### 4.2 Customer satisfaction with ATM banking services

Table 2 shows the status of customer satisfaction with using ATM banking services within the Commercial Bank of Ethiopia. The table shows that the highest mean score of all the customer satisfaction items is obtained and ranges from 3.83 to 4.11. According to Alhakimi and Alhariryb [55], the interpretations of the Likert scale results are: the mean score value of 1–2.32 indicates a low level, scores of 2.33–3.65 indicate a medium level, and scores of 3.66–5 indicate high level. In addition, the higher mean values (>3) refer to the customers are agreed on the item, and vice-versa. So, this result revealed that ATM users are more delighted with the ATM services they receive from the bank.

**Table 2:** Client Satisfaction with the ATM service quality of commercial bank of Ethiopia.

Items	Mean	Std. dev
I am happier with the ATM service quality than with ordinary banking service.	3.83	1.051
Your expectations before the use of ATM banking have been met currently.	3.98	1.190
I advise other consumers to utilize the ATM service	4.11	1.043
The introduction of ATMs has a positive effect in Banking practice.	4.05	1.098
**Overall**	**3.9913**	**.91005**

This signifies that the users of ATMs concur that ATM banking services offer higher satisfaction than conventional banking systems, have met customer expectations, offer adequate guidance on how to use and secure the ATM banking service, and, as a whole, have a more positive effect on banking practices than ordinary banking services. Overall, the grand mean for the overall satisfaction level is (3.9913) implying their satisfaction has reached a high level implying that the majority of users are satisfied with the provided ATM service of the Commercial Bank of Ethiopia in the study area and is evaluated as very good, though it needs an improvement on the different dimensions.

### 4.3 Effects of ATM service quality on customer satisfaction

The perception of the customer was analyzed based on the convenience, reliability, ease of use, fulfillment, and security dimension of ATM service quality offered by the Commercial Bank of Ethiopia. Table 3, shows the summary of customer perception of ATM services quality and the overall mean for the dimension of convenience, reliability, easiness, fulfillment, and security. As a result, the mean values of all dimensions vary from 2.37 to 4.18, showing that customers perceived that the ATM service is moderately convenient that there are enough ATMs, that offer 24/7 service, and that ATM waiting time for a given transaction is acceptable; reliable service that performs the service exactly as promised and completes the service correctly the first time; easy to use service that the ATM service is user friendly and uses simple and clear language; fulfillment that the service contains full banking service, provides information that exactly fits needs and the daily cash withdrawal limit of ATM is adequate; and finally, making transactions through ATM is safe, protects users privacy and transaction information and also has clear transaction safety policies regarding ATM followed by the bank. Moreover, the result established that the overall mean of all the ATM service quality dimensions from 2.41 to 3.98 referring that the ATM service their bank provides is convenient, reliable, easy to use, fulfilled, and secured in Commercial Bank of Ethiopia operated in the regional state of Afar.

**Table 3:** Customer perception of ATM service.

ATM Convenience	Mean	Std. dev
There are enough ATMs available to meet the demand of the populace.	2.67	1.337
The bank's ATM is accessible every day of the week, 24 h a day.	2.52	1.286
In the bank, the length of time an ATM user must wait to complete a particular transaction is acceptable.	2.45	1.248
**Overall**	**2.5482**	**1.16294**
**ATM Reliability**		
In the bank, ATM services are reliable enough that you don't need to carry cash wherever you go.	3.90	1.021
In the bank, ATM delivers the service exactly as promised.	3.87	1.046
In the bank, ATM completes the service right the first time.	3.66	1.197
**Overall**	**3.8083**	**.91341**
**ATM Ease to use**		
In the bank, ATM services are user-friendly	3.67	1.185
In the bank, ATMs provide tailored services for disabled persons.	3.39	1.281
In the bank, ATMs use simple and clear language.	3.60	1.326
**Overall**	**3.5520**	**1.12990**
**ATM Fulfillment**		
In the bank, ATMs contain full banking services.	4.02	1.265
In the bank, ATM banking provides information that exactly fits needs.	4.18	1.100
In the bank, the daily cash withdrawal limit of ATM is adequate	3.75	1.106
**Overall**	**3.9846**	**.99843**
**ATM Security/Privacy**		
In the bank, Making transactions through ATM is safe.	2.49	1.254
In the bank, ATMs protect my privacy and transaction information.	2.37	1.200
The bank has clear transaction safety policies regarding ATM	2.39	1.132
**Overall**	**2.4171**	**1.03639**

### 4.4 Measurement model assessment

Each latent construct was measured with multiple indicators to reach a high level of validity. The psychometric properties of the survey instrument were tested using CFA employing structural equation modeling (SEM) analysis with SPSS Amos version 23 to assess the quality of the measures. The measurement scales are provided in Table 4.

**Table 4:** Confirmatory factor analysis (CFA) and Convergent validity.

Constructs	Items	Factor Loading	CR	AVE	Cronbach α
ATM Convenience	CON 1: There are enough ATMs available to meet the demand of the populace.	.787			
	CON 2: The bank's ATM is accessible every day of the week, 24 h a day.	.947	.978	.722	.884
	CON 3: In the bank, the length of time an ATM user must wait to complete a particular transaction is acceptable.	.807			
ATM Reliability	REL 1: In the bank, ATM services are reliable enough that you don't need to carry cash wherever you go.	.694			
	REL 2: In the bank, ATM delivers the service exactly as promised	.754	.863	.551	.795
	REL 3: In the bank, the ATM completes the service right the first time.	.778			
ATM Ease to use	EOU 1: In the bank, ATM services are user-friendly	.862			
	EOU 2: In the bank, ATMs provide tailored services for disabled persons.	.826	.932	.698	.874
	EOU 3: In the bank, ATMs use simple and clear language.	.819			
ATM Fulfillment	FUL 1: In the bank, ATMs contain full banking services.	.738			
	FUL 2: In the bank, ATM banking provides information that exactly fits needs.	.846	.925	.625	.829
	FUL 3: In the bank, the daily cash withdrawal limit of ATM is adequate	.784			
ATM Security	SEC 1: In the bank, Making transactions through ATM is safe	.879			
	SEC 2: In the bank, ATMs protect my privacy and transaction information	.896	.952	.665	.832
	SEC 3: The bank has clear transaction safety policies regarding ATM service	.650			
Customer Satisfaction	SAT 1: I am happier with the ATM service quality than with ordinary banking service.	.705			
	SAT 2: Your expectations before the use of ATM banking have been met currently.	.676	.916	.609	.851
	SAT 3: I advise other consumers to utilize the ATM service	.840			
	SAT 4: The introduction of ATMs has a positive effect on Banking practice.	.882			

Tests of sampling adequacy were initially carried out before the analysis. Kaiser-Meyer-Olkin (KMO) statistic is 0.84, which is appropriately higher than the suggested cut cut-off of 0.60 [56]. Additionally, the Bartlett test of Sphericity (approximately: χ2 = 5264.913, df = 171, significance 0.000) was significant, at the 1% level.

Confirmatory factor analysis (CFA) was used to evaluate the measurement model. Based on the criteria of Cronbach's alpha and factor loading, the reliability of each construct was tested. For reliability to be considered acceptable, the Cronbach alpha and factor loading must surpass 0.70 and 0.50, respectively [57]. Table 4 presents the findings of the assessment of the measurement model and demonstrates that each variable's factor loading is greater than 0.6, and Cronbach alpha is greater than 0.70. Reliability has been determined to be at an acceptable level in this study. Opponents of Cronbach's α argue that while it is a straightforward reliability indicator based on internal consistency, it is ineffective for estimating mistakes brought on by variables outside of an instrument, such as variations in testing conditions or respondent characteristics over time [56]. Because they are more parsimonious than Cronbach's, composite reliability (CR) and average variance extracted (AVE) are suitable options for SEM [58]. As a result, a validity test consisting of convergent and discriminant validity was conducted to assess the reliability of the constructs and the items**.** Hair, Ringle [59] noted that the index of composite reliability (CR) should exceed 0.70 whereas the index of average variance extracted (AVE) should exceed 0.50 while examining convergent validity. According to Table 4, which lists the results for convergent validity, the values of CR, which range from 0.863 to 0.978, are greater than the 0.70 suggested threshold value. The findings based on factor loadings, Cronbach's alpha, and CR confirmed the five-factor structure as the dimensions of ATM service quality.

Convergent and discriminant validity were used to evaluate the measuring scale's validity. The degree to which latent variables have a significant amount of variance in common is measured by convergent validity [60]. The AVE in items by their respective constructs must be bigger than the variance unexplained for convergence validity to be attained (i.e. AVE >0.50). The findings in Table 4 showed that the constructs match the standards outlined by Ref. [57], with an AVE between 0.551 and 0.722 (>0.50). The study's convergent validity is therefore verified.

### 4.5 Structural model estimation

In this model, there were five exogenous variables (convenience, reliability, ease of use, fulfillment, and security/privacy), and one endogenous variable (satisfaction with service quality). The model was tested for good fit using various model fit indices and it was determined that the fit was adequate by the following standards: Chi-square (CMIN/DF) = 1.68; Goodness-of-Fit Index (GFI) = 0.90; Adjusted Goodness-of-Fit Index (AGFI) = 0.88; Incremental fit index (IFI) = 0.94; Tucker-Lewis Index (TLI) = 0.93; Comparative Fit Index (CFI) = 0.94; and Root Mean Square Error of Approximation (RMSEA) = 0.04. The findings of all goodness of fit indices met the specified levels from the literature (See Table 5), indicating that our data matched our model quite well.

**Table 5:** Model fit indices.

Model fit measures	CMIN/df	GFI	AGFI	IFI	TLI	CFI	RMSEA
Recommended value	<3^a^	>0.80^b^	>.80^c^	>0.90^c^	>0.90^c^	>0.90^c^	<0.08^d^
Model Value	1.68	0.90	0.88	0.94	0.93	0.94	0.04

a [ [58]].

b [ [61]].

c [ [58]].

d [ [62]].

**Fig. 2:**
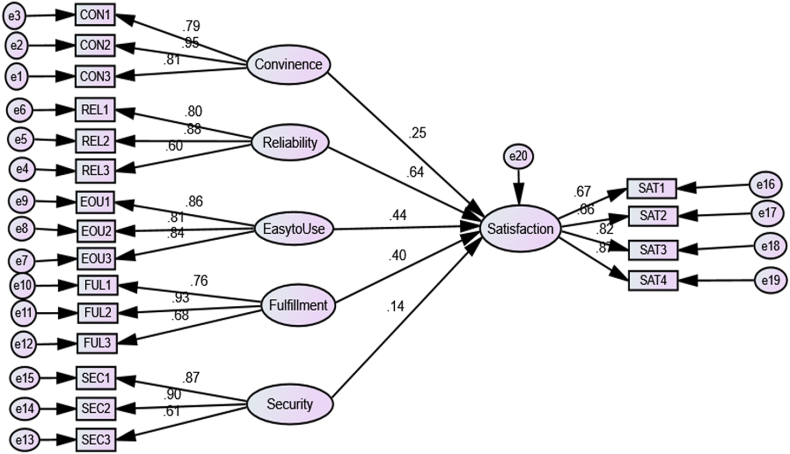
Path diagram of structural model.

The relation was developed and examined by the suggested model after the SEM model fit was determined**.** Fig. 2 depicts the predicted association. Out of the five ATM service quality aspects, all were found to be statistically significant, supporting four out of the five relationship hypotheses. The findings show that reliability, ease of use, fulfillment convenience, and security/privacy are the main predictors of customer satisfaction with ATM service quality. According to the path coefficients of the SEM results shown in Table 6, the reliability of the ATM service is the most predictor of customers' satisfaction (standardized beta = 0.636), followed by ease of use (standardized beta = 0.44), fulfillment (standardized beta = 0.40), convenience (standardized beta = 0.25), and security/privacy (standardized beta = 0.14), the factor that has the least contribution to the service quality of the ATM and customer satisfaction.

**Table 6:** Hypothesis testing.

Path	Coefficients	S.E.	C.R.	P	Results
Convenience → Customer Satisfaction	.247	.027	6.154	0.001	Supported
Reliability → Customer Satisfaction	.636	.043	10.962	0.001	Supported
Fulfillment → Customer Satisfaction	.399	.034	8.380	0.001	Supported
Security/Privacy → Customer Satisfaction	.142	.038	3.639	0.001	Unsupported
Easy to Use → Customer Satisfaction	.441	.030	9.173	0.001	Supported

## 5 Discussions

The purpose of the study was to identify the dimensions of ATM service quality and investigate the relationship between customer satisfaction and ATM service quality constructs. In this study, a five-factor structure was developed and tested, and the results showed that ATM quality is multidimensional, in line with recently published literature on ATM service quality [13,15,34]. The SEM approach discovered that reliability, ease of use, fulfillment, convenience, and security/privacy predicted customer satisfaction.

The study has confirmed that reliability is the major determinant of customer satisfaction with ATMs. The result of this study indicates that the dimension of reliability has a positive and significant association with customer satisfaction. This implies that ATM services are reliable enough that users don't need to carry cash wherever they go, deliver the service exactly as promised, and complete the service right the first time client will be more satisfied with ATM banking services. This finding is in line with the findings [3,10,17,19], which suggested that ATM service quality is an antecedent of customer satisfaction with a significant and positive influence on it.

The finding of this study also shows that the ease of use of ATM service quality dimension is an important contributor to customer satisfaction. Clients expect the ATM to be straightforward because even the most tech-savvy customers occasionally find technologies to be a little daunting. Easy-to-understand language, services adapted to the needs of people with disabilities, and user-friendly instructions were all deemed essential for enhancing the customer experience with ATMs. These results are consistent with the findings of other empirical studies such as [17,47,48,63].

In addition, this study further indicates that fulfillment of the ATM service is a good predictor of customer satisfaction. The outcome also implies that, if the ATM banking service daily cash withdrawal limit of ATM is adequate provides information that exactly fits needs, and contains full banking service the level of customer satisfaction could be improved. This finding is in tandem with the finding of [13,33].

Similarly, the convenience of ATM service quality is another significant dimension of ATM service quality in predicting customer satisfaction. This denoted that if the banks install other ATMs at different locations like shopping areas, hotels, hospitals, college campuses, etc., enabling customers can carry out their banking activities whenever they want in a 24/7 h service, ATM banking service would be more convenient, and higher the customer satisfaction is likely to be. This finding is concurrent with the findings of [10,17,47,63].

Finally, the finding of this study indicates that the security or privacy of the ATM banking service to the customer has a positive and significant influence on customer satisfaction. This suggested that as the service secures the privacy of the customer, protects the customer's banking information, and has clear transaction safety policies will significantly improve customer satisfaction with the bank service. The result of the study is consistent with the works of [10,63,64]. Thus, to summarize the findings of the study reveal that convenience, reliability, ease of use, fulfillment, and security dimensions of ATM service, have a positive and significant effect on overall ATM service quality in Commercial Bank of Ethiopia in the Afar regional state.

## 6 Conclusion, theoretical and practical implications of the study

The purpose of the study was to examine the effect of ATM service quality on customer satisfaction in the case of the Commercial Bank of Ethiopia. The study used primary data for analysis, which were obtained from 346 ATM users in the region selected area, to achieve this purpose. The required data are collected in person through a structured questionnaire. After data collection, the study employed descriptive and econometric data analysis methods. The findings of this study obtained from the descriptive analysis showed that the majority of current ATM users are youth between the ages of 18–35, gender-wise the males are the dominant users, occupationally salaried and unemployed are the majority users, and businessmen/women and students are not an active participant in using the service, educational level diploma and first-degree holders are the majority users, as indicated by the overall mean of all ATM service quality dimensions, which runs from 3.21 to 3.63 that the customers agreed that the ATM service is more reliable, easy to use, fulfilled, convenient, and secured provided by the bank in the study area.

Moreover, the results obtained from the SEM revealed that reliability, ease of use, fulfillment, convenience, and security of ATM service quality dimensions have a positive and significant contribution to customer satisfaction. Out of this reliability has been identified as the key factor in predicting customer satisfaction.

### 6.1 Theoretical implication

The study contributes to the existing literature by identifying five dimensions of ATM service quality (reliability, ease of use, fulfillment, convenience, and security/privacy). This helps researchers and practitioners to have a comprehensive understanding of the key factors that influence customers' perception of ATM services. The research findings in the study contribute to the current understanding of the topic by establishing the connection between the quality of ATM services and customer satisfaction. The study emphasizes the importance of these aspects in determining overall customer satisfaction. It presents arguments on why and how the quality of ATM services influences customer satisfaction, thereby adding to the existing literature on this relationship. Through the use of structural equation modeling (SEM), the study ensures the reliability and validity of the developed five-factor structure. This finding contributes to the methodological aspect of assessing service quality dimensions.

### 6.2 Practical implications

The findings of this study provide valuable insights for ATM service providers and managers in identifying areas that need improvement. By focusing on improving the identified dimensions of ATM service quality (reliability, convenience, ease of use, fulfillment, and security/privacy), ATM service providers can enhance customer satisfaction. This may involve enhancing system reliability, simplifying the user interface, ensuring prompt transaction processing, increasing convenience through additional functionalities, and prioritizing security and privacy measures. Implementing findings from this study enables organizations to align their strategies with customer expectations and requirements. Understanding the dimensions that drive customer satisfaction empowers service providers to develop targeted initiatives, such as training programs for employees, technological advancements, and personalized customer experiences, to improve overall service quality. Differentiating ATM services based on the identified dimensions can provide a competitive advantage for banks. By excelling in reliability, ease of use, fulfillment, convenience, and security/privacy of ATM services, institutions can attract and retain satisfied customers who perceive their ATM services as superior to competitors'. This can ultimately lead to increased market share and customer satisfaction.

Overall, this study provides theoretical insights into the dimensions of ATM service quality and its relationship with customer satisfaction. It also offers practical implications for banks, financial institutions, and ATM manufacturers to enhance service quality, and improve customer satisfaction in the ATM domain.

### 6.3 Limitations and future research directions

Although the research paper has greatly enhanced our comprehension and assessment of ATM service quality, it is crucial to recognize its inherent limitations. One major limitation is that the study was conducted specifically in one city and two towns of the Afar region, focusing on ATM users of the Commercial Bank of Ethiopia. The attitudes and experiences of customers from different provinces or banks may vary, and therefore, the results may only provide limited insights into customer attitudes toward ATM service quality and its impact on customer satisfaction. To address this limitation, additional research should be conducted to investigate the behavior of customers located in different regions.

Furthermore, since this study solely relied on quantitative methods for data collection through surveys, it is important to acknowledge the limitations associated with this approach. Future research should consider employing mixed methodology and qualitative approaches to gain a more comprehensive understanding of the topic. Additionally, it is recommended that future studies explore the relationship between other dimensions of service quality and customer satisfaction, loyalty, and retention in various other self-service technologies such as Internet banking and mobile banking. Comparing the cross-cultural service quality of conventional commercial banks would also be of interest in future research.

## Author contribution statement

Abibual Getachew Nigatu; Atinkugn Assefa Belete; Getnet Mamo Habtie: Conceived and designed the experiments; Performed the experiments; Analyzed and interpreted the data; Contributed reagents, materials, analysis tools or data; Wrote the paper.

## Funding statement

During this investigation, there was no financial support.

## Data availability statement

Data will be made available on request.

## Declaration of interest's statement

The authors declare no conflict of interest.

## Declaration of competing interest

The authors declare that they have no known competing financial interests or personal relationships that could have appeared to influence the work reported in this paper.
