# Supplementary material for: Effects of automated teller machine service quality on customer satisfaction: Evidence from commercial bank of Ethiopia
Source: Heliyon. 2023 Aug 14;9(8):e19132. doi: 10.1016/j.heliyon.2023.e19132 (PMC10457530; doi:10.1016/j.heliyon.2023.e19132)
Supplement: Multimedia component 1 [file mmc1.docx]

**Questionnaire completed by ATM card users**

**Dear respondents**

The main purpose of this study in general and questionnaire in particular is to examine **“Effects of Automated Teller Machine Service on Customer Satisfaction"**. Hence, I kindly request you to fill the questionnaire carefully and at your best knowledge in all regard to meet the aim of the study. Your response are highly honored and kept confidential.

You may contact the first researcher with the following name and address

Name: Abibual Getachew Telephone: +251922913038 Email: **Abibual8@jmail.com**

**General Instructions**

- Your participation is voluntary.
- You don’t need to write your identification like name and cell phone.
- Please simply tick (
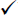
 ) on the appropriate box.
- ***I thank you in advance for your kind cooperation***

**General questions:**

***Please put right mark (
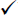
) in front of your choice box that express yourself.***

1. **Gender:** a. Male b. Female
2. **Age :** a. 18- 26 b. 27-35 c. 36-50 d. 50 and above
3. **Marital status**: a. Single b. Married c. Divorced d. Widowed
4. **Level of education:** a. Illiterate b. Primary education

c. Secondary education d. Diploma and above

5. **Occupation:** a. Student b. Unemployed c. Salaried d. Businessman

6. How long you have been the customer of CBE bank?

a. below 1 year b. 1-2 years c. 3-4 years d. 5-6 years e. Over 6 years

7. Number of years you have been using ATM banking service

a. below 1 year b. 1-2 years c. 3-4 years d. 5-6 years e. Over 6 years

The following questions are related with ATM banking service quality of commercial bank of Ethiopia. Please tick (√) the box that represents your agreement or disagreement with each statement.

| **No.** | **Dimension and its items** | Strongly  disagree  (1) | disagree  (2) | Neutral  (3) | Agree  ( 4) | Strongly  agree  (5) |
| --- | --- | --- | --- | --- | --- | --- |
|  | **ATM Convenience** |  |  | | | |
|  | In the commercial bank of Ethiopia, there are enough ATMs available to meet the demand of the populace. |  |  |  |  |  |
|  | The bank's ATM is accessible every day of the week, 24 hours a day. |  |  |  |  |  |
|  | In the bank, the length of time an ATM user must wait to complete a particular transaction is acceptable. | (1) | (2) | (3) | (4) | (5) |
|  | **ATM Reliability** | | | | | |
|  | In the commercial bank of Ethiopia, ATM services are reliable enough that you don’t need to carry cash wherever you go. |  |  |  |  |  |
|  | In the bank, ATM delivers the service exactly as promised. |  |  |  |  |  |
|  | In the bank, ATM completes the service right the first time. |  |  |  |  |  |
|  | **ATM Ease to use** | | | | | |
|  | In the commercial bank of Ethiopia, ATM services are user friendly. |  |  |  |  |  |
|  | In the bank, ATMs provide tailored services for disabled persons. |  |  |  |  |  |
|  | In the bank, ATMs use simple and clear language. |  |  |  |  |  |
|  | **ATM Fulfillment** | | | | | |
|  | In the commercial bank of Ethiopia, ATMs contain full banking services. |  |  |  |  |  |
|  | In the bank, ATM banking provides information that exactly fits needs. |  |  |  |  |  |
|  | In the bank, the daily cash withdrawal limit of ATM is adequate. |  |  |  |  |  |
|  | **ATM Security/Privacy** | | | | | |
|  | In the Commercial Bank of Ethiopia, Making transactions through ATM is safe. |  |  |  |  |  |
|  | In the bank, ATMs protect my privacy and transaction information. |  |  |  |  |  |
|  | The bank has clear transaction safety policies regarding ATM. |  |  |  |  |  |
|  | **Client satisfaction with ATM services** | | | | | |
|  | I am happy with the ATM service of commercial bank of Ethiopia than ordinary banking service. |  |  |  |  |  |
|  | Your expectations before the use of ATM banking have been met currently. |  |  |  |  |  |
|  | I advise my friends and families to utilize the ATM service provided by CBE. |  |  |  |  |  |
|  | The introduction of ATMs has a positive effect in Banking practice. |  |  |  |  |  |

**Thank you for your cooperation!**
